# Discriminatory Molecular Biomarkers of Allergic and Nonallergic Asthma and Its Severity

**DOI:** 10.3389/fimmu.2019.01051

**Published:** 2019-05-09

**Authors:** Selene Baos, David Calzada, Lucía Cremades-Jimeno, MªÁngeles de Pedro, Joaquín Sastre, César Picado, Joaquín Quiralte, Fernando Florido, Carlos Lahoz, Blanca Cárdaba

**Affiliations:** ^1^Immunology Department, IIS-Fundación Jiménez Díaz-UAM, Madrid, Spain; ^2^CIBERES, CIBER of Respiratory Diseases, Madrid, Spain; ^3^Allergy Department, Fundación Jiménez Díaz Hospital, Madrid, Spain; ^4^Pulmonology Department, Clinic de Barcelona Hospital, Institut d'Investigacions Biomèdiques August Pi iSunyer, Barcelona, Spain; ^5^Allergy Department, Vírgen del Rocío University Hospital, Seville, Spain; ^6^Allergy Department, San Cecilio University Hospital, Granada, Spain

**Keywords:** asthma, biomarkers, gene expression, protein expression, allergy

## Abstract

Asthma is a complex disease comprising various phenotypes and endotypes, all of which still need solid biomarkers for accurate classification. In a previous study, we defined specific genes related to asthma and respiratory allergy by studying the expression of 94 genes in a population composed of 4 groups of subjects: healthy control, nonallergic asthmatic, asthmatic allergic, and nonasthmatic allergic patients. An analysis of differential gene expression between controls and patients revealed a set of statistically relevant genes mainly associated with disease severity, i.e., *CHI3L1, IL-8, IL-10, MSR1, PHLDA1, PI3*, and *SERPINB2*. Here, we analyzed whether these genes and their proteins could be potential asthma biomarkers to distinguish between nonallergic asthmatic and asthmatic allergic subjects. Protein quantification was determined by ELISA (in serum) or Western blot (in protein extracted from peripheral blood mononuclear cells or PBMCs). Statistical analyses were performed by unpaired *t*-test using the Graph-Pad program. The sensitivity and specificity of the gene and protein expression of several candidate biomarkers in differentiating the two groups (and the severity subgroups) was performed by receiver operating characteristic (ROC) curve analysis using the R program. The ROC curve analysis determined single genes with good sensitivity and specificity for discriminating some of the phenotypes. However, interesting combinations of two or three protein biomarkers were found to distinguish the asthma disease and disease severity between the different phenotypes of this pathology using reproducible techniques in easy-to-obtain samples. Gene and protein panels formed by single biomarkers and biomarker combinations have been defined in easily obtainable samples and by standardized techniques. These panels could be useful for characterizing phenotypes of asthma, specifically when differentiating asthma severity.

## Introduction

The heterogeneity of the clinical phenotypes in asthma and respiratory allergy shows the complexity of this type of diseases, and this reflects the involvement of genetic and environmental factors, and therefore, its multifactorial character. Many investigations have aimed to identify the environmental risk factors and genes associated with these diseases (1). Faced with such great complexity, new techniques such as massive analysis or *omics* have become important tools in efforts to advance existing knowledge of the diagnosis, follow-up, and treatment-monitoring of asthmatic, and allergic diseases, and also in the search for risk-related or protective biomarkers and the development of new drugs (2).

Precision medicine, an increasingly important approach to the treatment and prevention of disease, has been defined by the U.S. National Library of Medicine as a strategy that takes into account the variability in genes, in the environment, and in individual lifestyles. The end goal of precision medicine is to find predictive parameters with which to select the most appropriate prophylactic or therapeutic strategy for a given disease within a specific group of patients (3). Precision medicine implicates that individuals are classified as subpopulations that differ in their susceptibility to a certain disease. To identify subpopulations of patients or phenotypes (4), the approach of precision medicine is based in the underlying mechanisms of the distinct forms of each disease, that is, endotypes (5), using related measures that act as biomarkers (6).

From a clinical point of view, asthma is a heterogeneous disease usually characterized by chronic airway inflammation with a great number of different phenotypes. Despite its clinical complexity, most efforts to find new treatments for asthma have centered on allergic asthma or asthma mediated by type 2 inflammation, which is responsible for disease in 50–80% of asthmatic patients. These patients would be classified in the so called type T2 or T2-high endotype. Type 2 immune response has been extensively characterized and defined as an increase of T-helper 2 (Th2) cytokines, mainly of IL4, IL5, and/or IL13 likely derived from both adaptive (mainly Th2 lymphocytes), and innate, mainly innate lymphoid cells type 2 (ILC2) immune cells resulting in eosinophilic airway infiltration. These patients are allergic subjects with high total IgE levels and high eosinophil counts. In comparison, 10–33% of subjects with asthma have no associated allergy (nonallergic asthma), with a non-type 2 inflammation (non-T2 or T2-low endotype) (7), and the mechanisms that contribute to the immune response are less clear in these subjects. In many cases, instead of an eosinophilic inflammation, there exists a prevalence of neutrophils (8–12). To date, no directed therapy has been found to be effective against this endotype (13).

The type-T2 biomarkers used in clinical practice are limited to eosinophil counts in sputum and blood, FeNO (fractional concentration of exhaled nitric oxide), and IgE levels in serum. In addition to these markers, however, periostin is becoming increasingly relevant in clinical practice, this despite the findings of Korevaar et al., who showed that in severe asthma, there is a poor correlation between the number of eosinophils in blood, FeNO, periostin, and serum IgE levels on the one hand and the number of eosinophils in sputum (14). Therefore, conventional biomarkers of T2-high asthma show limited sensitivity and specificity, and potential biomarkers are still not available for routine use in clinical practice due to a lack of validation and standardization. The T2-low endotype has been less exhaustively characterized. It is associated with airway neutrophilia and steroid insensitivity to airway obstruction (15, 16). Some proposed biomarkers for this endotype include neutrophilia in blood and sputum (17), serum IL-6, IL-8 in sputum, and neutrophil elastase protein (18), though all have numerous limitations. These novel biomarkers are targeted to neutrophilic inflammation that can be originated by other causes apart from asthma disease, for instance by high dosage of corticosteroid medication (19), exposure to pollution, cigarette smoke and bacterial infection. In a recent work, new gene and protein biomarkers *CHI3L1, IL-8, IL-10, MSR1, PHLDA1, PI3*, and *SERPINB2*, were proposed to discriminate healthy control subjects from nonallergic asthmatic patients (T2-low) and to measure asthma severity (20). The relevance of these potential biomarkers in asthma and allergy diseases was extensively discussed (21).

Thus, there remains an unmet clinical need in the study of the mechanisms and biomarkers for both T2-high and T2-low endotypes as concerns their ability to predict response to targeted therapy (22). The development of solid biomarkers combined with a deeper understanding of the pathology and physiology of asthma will allow a more direct and effective treatment of asthmatic patients through approaches based on precision medicine (23). In this work, we seek to corroborate at genetic and protein level the implication of previously described biomarkers associated with allergic (T2-high) and nonallergic asthma (T2-low or non T2) diseases in order to define their potential as diagnostic and disease-severity biomarkers.

## Materials and Methods

### Subjects

The study population comprised 60 unrelated subjects previously described by Baos et al. (21): 30 patients with nonallergic asthma (NA) and 30 patients with allergic asthma (AA). Different biological samples (PBMCs and sera) were obtained from the asthma biobank of the CIBERES (*Ciber de Enfermedades Respiratorias*) located at the IIS-*Fundación Jiménez D*í*az*-UAM in Madrid (IIS-FJD-UAM) (24). Patients were diagnosed as having severe, moderate, or mild asthma according to the Spanish Guidelines for the Management of Asthma or GEMA (25). Daily mean inhaled corticosteroids administration over the 6 months preceding diagnosis and during sample collection was 1,488 ± 541 μg in severe, 1,100 ± 977.75 μg in moderate, and 450 ± 463.68 μg in mild asthmatic patients. On the day of sample collection, the subjects did not take systemic medication of any kind. Pulmonary function tests were carried out and different parameters were determined, i.e., the predicted percentage of forced vital capacity (% FVC), forced expiratory volume in 1 s (% FEV_1_), and the post bronchodilator test (% PBD) or reversibility test. All subjects were tested by skin prick test against a panel of common allergens, including mites (*Dermatophagoides pteronyssinus, Dermatophagoides farinae*, and *Lepidoglyphus destructor*), epithelia (cat and dog), cockroaches (*Blatella orientalis* and *Blatella germanica*), pollens (*Cypress, banana shadow*, olive, mixture of grasses, *Artemisia, Parietaria*, and *Salsola*), and fungi (*Alternaria, Cladosporium, Aspergillus*, and *Penicillium*). Written informed consent was obtained from each subject in accordance with the Declaration of Helsinki. Ethical approval for the study was obtained from the ethics and research committees of the participating hospitals.

### Peripheral Blood Mononuclear Cell Isolation and Protein Extraction

Peripheral blood mononuclear cells (PBMCs) were isolated from heparin-containing peripheral blood samples by gradient centrifugation using Lymphoprep (Comercial Rafer, Zaragoza, Spain) following the manufacturer's instructions. PBMCs were isolated in sterile conditions using endotoxin-free reagents. RNA and total protein were isolated from PBMCs (10^6^ cells) using the Trizol method (Invitrogen, Carlsbad, CA, USA). RNA was quantified and its purity was checked by spectrophotometry using the nanodrop (ND-1000 Spectrophotometer) system. Protein levels were quantified by applying the BCA method (Thermo Fisher Scientific, Rockford, IL, USA).

### Differential Gene Expression by qRT-PCR

Gene expression analyses were performed by qRT-PCR using microfluidic cards following the protocols described by Baos et al. (21).

### Gene Selection

*CPA3, CHI3L1, IL-1R2, IL-8*, and *PI3* were selected as candidate biomarkers for both the NA and AA groups (significance established at relative gene quantification of higher than 4 or lower than 0.25 compared to the healthy control group) (24), as these were the most relevant genes in the two asthma groups. Genes that did not meet the strict criteria (RQ > 4 or <0.25) in all of the comparisons (24) were not taken into account for the protein expression analysis. In addition to this criterion, the proteins CHI3L1, IL-8, IL-10, PI3, PHLDA1, and SERPINB2, which were selected to compare the NA and AA groups, were previously found to be the most relevant genes in the NA group (21).

### Soluble Protein Level Analysis of IL-8, IL-10, CHI3L1, PI3, and POSTN

Using a commercially available ELISA kit, soluble biomarkers were quantified in all the study subjects. Levels of CHI3L1, IL-8, IL-10, PI3, and POSTN were measured in the subjects' serum using the human ELISA kits manufactured by R&D Systems (Minneapolis, MN, USA) for CHI3L1, PI3, and POSTN; by ImmunoTools (Friesoythe, Germany) for IL-10; and by Diaclone (Besancon Cedex, France) for IL-8. The procedure was carried out in accordance with each manufacturer's protocol. *POSTN*, or periostin, was analyzed at the protein level given its relevance as a protein associated with allergic asthma (26, 27).

### Protein Expression Analysis of PHLDA1 and SERPINB2

Protein determination of PHLDA1 and SERPINB2 was performed by Western blot as these were not soluble proteins or no commercial ELISA kit was available at the time. They were quantified in the total protein extracted from PBMCs. PHLDA1 was studied in 5 NA (3 severe and 2 moderate-mild) and 6 AA subjects (3 severe and 3 moderate-mild), and we studied SERPINB2 in 11 NA (6 with severe asthma and 5 with moderate-mild diagnosis), and 11 AA subjects (6 with severe asthma and 5 with moderate-mild diagnosis). We used the Western blot procedure pertaining to the Invitrogen Western Breeze® Chemiluminescent Western Blot Immunodetection Kit (Life Technologies, Carlsbad, CA, USA) as previously described (24). PHLDA1 was detected with a rabbit anti-human polyclonal PHLDA1 antibody (Thermo Fisher Scientific) at a 1:500 dilution and SERPINB2 with the rabbit anti-human polyclonal SERPINB2 antibody by R&D Systems at a dilution of 1:250. The result was visualized using a luminescent image analyzer: the ImageQuant LAS 4000 (GE Healthcare Life Science, Little Chalfont, Buckinghamshire, UK). Data from specific protein results were relative to β-Actin (dilution 1:1000; Cell Signaling Technology, Danvers, MA, USA) expression.

### ROC Curve Analysis at the Gene and Protein Level

Sensitivity *vs*. specificity on ROC curve plots and the area under the curve (AUC) are effective measures of accuracy when evaluating the diagnostic ability of tests to discriminate the true state of subjects, finding optimal cut-off values. A ROC curve was constructed for the candidate biomarkers common to the NA and AA groups, examining severity and total expression at the gene level. At the protein level, CHI3L1, IL-8, IL-10, SERPINB2, PHLDA1, PI3, and POSTN were studied, as these were found to be the most relevant in previous research (20, 21). Four kinds of comparisons were performed: total NA group vs. total AA group, severe NA patients vs. severe AA patients, moderate-mild NA patients vs. moderate-mild AA patients, and severe AA patients vs. moderate-mild AA patients. Comparison between severe NA patients vs. moderate-mild NA patients has already been described by Baos et al. (20). A guide for interpreting the ROC curves has been previously described (20). Only the results with a 95% confidence interval (95% CI) between 0.70 and 1 were considered statistically significant.

### Statistical Analysis

The levels and relative expression of the proteins studied were compared between groups by unpaired *t*-test, using the Graph-Pad InStat 3 program. Statistical significance was established at a two-tailed *P*-value < 0.05. The ROC curve analyses were performed using the R program.

## Results

### Subjects

The study population has been described previously (21), and Table 1 summarizes the demographic and clinical parameters of the two groups studied (Table 1A). The NA patients were significantly older than the AA subjects (58.03 ± 13.14 vs. 42.37 ± 15.44 years, respectively; *P* <0.0001). There were more women than men in both groups and in a similar proportion. Smoking habits were similar in both groups. The NA group presented no allergic symptoms, with negative results on the skin prick test against a panel of common allergens. In contrast, the AA patients were all allergic to airborne allergens. Mean levels of total IgE were significantly higher in the AA group (371.64 ± 437.69 IU/ml) compared to the NA patients (82.04 ± 80.63 IU/ml) (*P* < 0.001). The concomitant diseases found in the NA group were as follows: nonatopic rhinitis (80%), sinusitis (50%), polyposis (43.3%), esophageal reflux (20%), eczema (6.67%), and rash (6.67%); and in the AA group, rhinitis (90%), esophageal reflux (50%), sinusitis (26.67%), rash (16.67 %), polyposis (10%), and eczema (3.33%). Each group contained 50% severe asthma patients, and the other 50% moderate-mild asthma subjects. The %FEV_1_ and %FVC values were similar. When these parameters were analyzed according to disease severity (Table 1B), significant differences were found only in the NA group (%FEV_1_: 66.33 ± 16.62 severe patients vs. 85.38 ± 21.03 moderate-mild patients, *P* = 0.0127; %FVC: 69.93 ± 19.94 severe patients vs. 94 ± 19.52 moderate-mild patients, respectively, *P* = 0.0031). The percentage and number of eosinophils in the NA group were normal (3.83 ± 2.24 vs. 273.86 ± 137.13 cells/μl) (cut-off: 1–4% and 50–450 cells/μl). No significant differences were found in the presence of eosinophils between severe and moderate-mild NA subjects (percentage: 3.73 ± 2.48 vs. 4.22 ± 1.25%, respectively; number: 264 ± 152.96 vs. 310 ± 52.57 cells/μl, respectively). In contrast, eosinophil presence was high among AA patients, with a percentage of 5.70 ± 3.40% and a count of 625.60 ± 999.28 cells/μl. No significant differences were observed when comparing the severe and moderate-mild AA patients (percentage: 5.89 ± 3.70 vs. 5.28 ± 2.85%, number: 739.50 ± 1180.74 vs. 359.83 ± 220.55 cells/μl, severe AA vs. moderate-mild AA, respectively) (Table 1B).

**Table 1A:** Characteristics of the study population.

	**N**	**Sex**	**Age**	**Smoking**	**Clinical diagnosis**		**Total IgE (IU/ml)**	**%FVC**	**%FVE_**1**_**	**%Eosinophils**	**Eosinophil count** **(cells/μl)**
**(A) CHARACTERISTICS OF THE GLOBAL POPULATION**											
Nonallergic asthmatic subjects (NA)	30	73.33% women 26.67% men	58.03 ± 13.14^*^	73.33% non-smokers 10% smokers 16.67% ex-smokers	50% severe asthma 30% moderate asthma 20% mild asthma	No allergic symptoms	82.04 ± 80.63	81.11 ± 22.69	75.18 ± 22.82	3.83 ± 2.24	273.86 ± 137.13
Allergic asthmatic subjects (AA)	30	80% women 20% men	42.37 ± 15.44^*^	68.97% non-smokers 6.90% smokers 24.14% ex-smokers	50% severe asthma 26.7% moderate asthma 23.3% mild asthma	43.3% allergic to pollen 56.7% not allergic to pollen	371.64 ± 437.69^#^	75.53 ± 16.58	73.33 ± 16.28	5.70 ± 3.40	625.60 ± 999.28

*% FVC, percentage of predicted value of forced vital capacity; % FEV_1_, percentage of predicted value of forced expiratory volume in 1 s*.

* *Statistically significant comparison (P < 0.0001) between the NA and AA group*.

# *Statistically significant comparison (P < 0.001) between the NA and AA group*.

**Table 1B:** Characteristics of the study population.

	**N**	**Total IgE (IU/ml)**	**% FVC**	**% FVE_**1**_**	**% Eosinophils**	**Eosinophils count (cells/μl)**
**(B) CHARACTERISTICS OF THE POPULATION ACCORDING TO ASTHMA SEVERITY**						
Severe NA subjects	15	88.85 ± 61.57	69.93 ± 19.94	66.33 ± 16.62	3.73 ± 2.48	264.00 ± 152.96
Moderate/mild NA subjects	15	75.24 ± 97.87	94.00 ± 19.52^#^	85.38 ± 21.03^*^	4.22 ± 1.25	310.00 ± 52.57
Severe AA subjects	15	306.27 ± 204.53	71.93 ± 12.38	69.27 ± 13.36	5.89 ± 3.70	739.50 ± 1180.74
Moderate/mild AA subjects	15	417.13 ± 583.84	79.13 ± 19.70	77.40 ± 18.31	5.28 ± 2.85	359.83 ± 220.55

*NA, nonallergic asthmatic; AA, allergic asthmatic; % FVC, percentage of predicted value of forced vital capacity; % FEV1, percentage of predicted value of forced expiratory volume in 1 second*.

* *Statistically significant comparison (p < 0.05) between the severe NA and moderate/mild NA group*.

# *Statistically significant comparison (p < 0.005) between the severe NA and moderate/mild NA group*.

### ROC Curve Analysis of Gene Expression

The five genes studied were classified into five categories (20), as seen in Table 2. When comparing the two groups of patients, *CPA3* (AUC value: 0.77) was considered a good biomarker for their differentiation. The rest (*CHI3L1, IL-1R2, IL-8*, and *PI3*) were classified as regular. To discriminate patients with severe disease in the two asthma groups, *PI3* (AUC value: 0.78) was seen to be a good biomarker. The gene expression of *CPA3* and *IL-8* (AUC value: 0.89 and 0.87, respectively) were effective biomarkers for distinguishing moderate-mild NA subjects from moderate-mild AA patients. When comparing the severe and moderate-mild AA groups, neither of the genes were considered good, as was observed in the severe NA vs. moderate-mild NA comparison (20).

**Table 2:** Classification of biomarkers by receiver operating characteristic (ROC) curves analysis at the genetic level.

**Comparison/AUC value**	**Excellent** **(AUC: 0.98–1)**	**Very Good** **(AUC: 0.91–0.97)**	**Good** **(AUC: 0.76–0.90)**	**Regular** **(AUC: 0.61–0.75)**	**Poor** **(AUC: 0.50–0.60)**
Total NA vs. Total AA			*CPA3*: 0.77	CHI3L1: 0.61 IL-1R2: 0.74 IL-8: 0.72 PI3: 0.74	
Severe NA vs. severe AA			*PI3*: 0.78	*CPA3*: 0.63 *IL-1R2*: 0.73 *IL-8*: 0.61	*CHI3L1* < 0.50
Moderate-mild NA *vs*. moderate-mild AA			*CPA3*: 0.89 *IL-8*: 0.87	*CHI3L1*: 0.74 *IL-1R2*: 0.71 *PI3*: 0.73	
Severe AA vs. moderate-mild AA				*CHI3L1*: 0.69 *CPA3*: 0.67 *IL-1R2*: 0.65 *IL-8*: 0.63	*PI3*: 0.50

*Summary of the ROC curve analysis of the gene biomarkers common to both asthma groups. AUC, area under the curve. ND, not determined due to a lack of data; NA, nonallergic asthma; AA, allergic asthma*.

### Protein Expression Analysis

**Figure 1:**
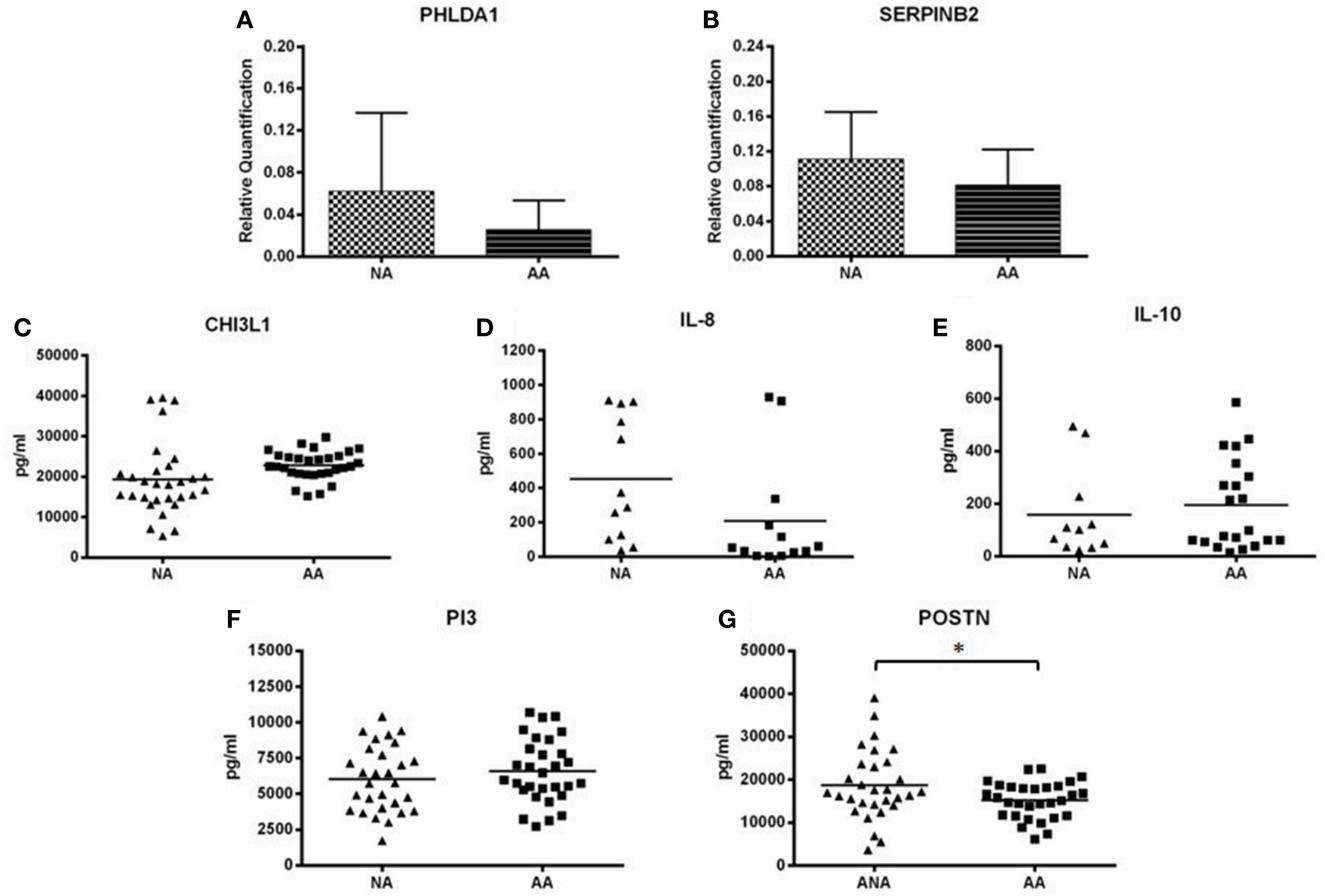
Mean levels of protein expression. **(A)** Mean levels of PHLDA1. **(B)** Mean levels of SERPINB2. **(C)** Mean levels of CHI3L1. **(D)** Mean levels of IL-8. **(E)** Mean levels of IL-10. **(F)** Mean levels of PI3. **(G)** Mean levels of POSTN. *Statistically significant comparison (*P* < 0.05) between the indicated groups. Protein levels of PHLDA1 and SERPINB2 were measured by Western blot in 5 NA and 6 AA subjects, and 11 NA and 11 AA, respectively. Densitometric analysis was done in individual blots (see “Materials and Methods”) using the β-actin protein for normalization. CHI3L1, IL-8, IL-10, PI3, and POSTN were quantified by ELISA in all patients included in the study population. The levels and relative expression of the proteins studied were compared among groups by unpaired *t*-test, using the Graph-Pad InStat 3 program. The error bars indicate the standard deviation. NA, total nonallergic asthma group; AA, total allergic asthma group.

**Figure 2:**
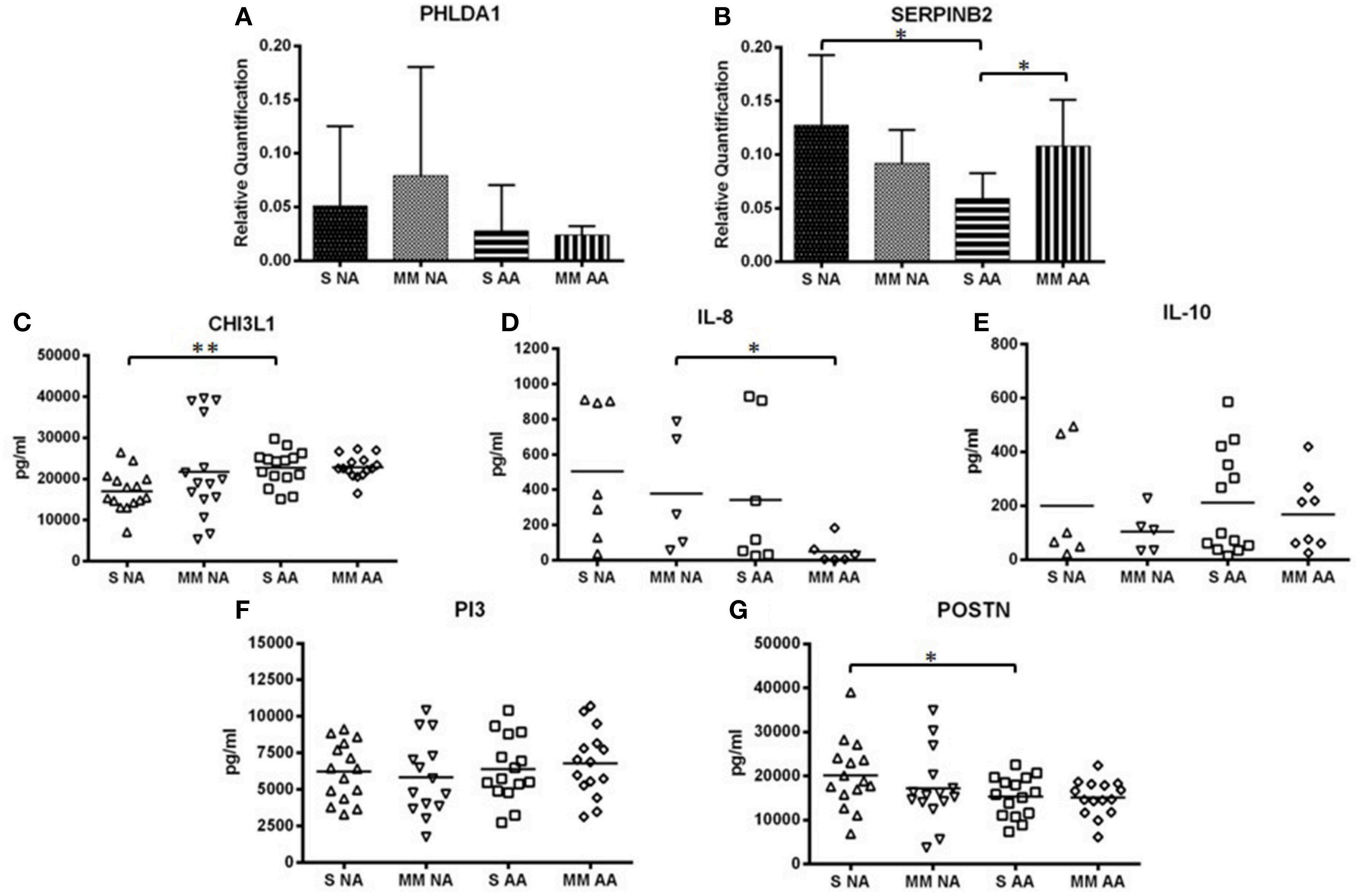
Mean levels of protein expression by asthma severity subgroup. **(A)** Mean levels of PHLDA1. **(B)** Mean levels of SERPINB2. **(C)** Mean levels of CHI3L1. **(D)** Mean levels of IL-8. **(E)** Mean levels of IL-10. **(F)** Mean levels of PI3. **(G)** Mean levels of POSTN. *Statistically significant comparison (*p* < 0.05) between the indicated groups. **Statistically significant comparison (*p* < 0.005) between the indicated groups. Protein levels of PHLDA1 were measured by Western blot in 3 severe NA and 2 moderate-mild NA patients, 3 patients with severe AA, and 3 with moderate-mild AA. Protein levels of SERPINB2 were measured by Western blot in 6 severe NA and AA subjects and in 5 moderate-mild NA and AA patients. Densitometric analysis was done in individual blots (see “Materials and Methods”) using the β-actin protein for normalization. CHI3L1, IL-8, IL-10, PI3, and POSTN were quantified by ELISA in all patients studied. The levels and relative expression of the proteins studied were compared among groups by unpaired *t*-test, using the Graph-Pad InStat 3 program. The error bars indicate the standard deviation. S, group of subjects with severe asthma; MM, group of subjects with moderate-mild asthma.

Figures 1, 2 provide a summary of the relative quantification (RQ) of the protein expression of PHLDA1 and SERPINB2 and mean sera levels (pg/ml) of CHI3L1, IL-8, IL-10, PI3, and POSTN. The study of CHI3L1, PI3, and POSTN gave detectable protein levels in all of the assays performed. However, we found a 53.33 and 63.3% of undetected values of IL-8 and IL10 in samples of NA patients and 56.67 and 30% in AA patients, respectively.

The protein level of POSTN was significantly higher in the NA subjects (18,679.59 ± 8,086.07 pg/ml) when compared to the AA subjects (15,199.93 ± 4,263.88 pg/ml) (*P* = 0.0415) (Figure 1G). Though the results were not statistically significant, the protein expression of PHLDA1 (NA patients: 0.062 ± 0.064 RQ; AA patients: 0.025 ± 0.027 RQ) (Figure 1A), SERPINB2 (NA patients: 0.11 ± 0.05 RQ; AA patients: 0.08 ± 0.04 RQ)(Figure 1B), and IL-8 (NA patients: 452.28 ± 357.72 pg/ml; AA patients: 207.02 ± 328.90 pg/ml) (Figure 1D) were higher in the NA group than in the AA group. CHI3L1 (Figure 1C) and IL-10 (Figure 1E) showed higher levels in the AA group (CHI3L1, AA vs. NA group: 22,812.55 ± 3,573.46 pg/ml vs. 19,364.77 ± 9,046.25 pg/ml, respectively; IL-10, AA vs. NA group: 194.96 ± 171.81 pg/ml vs. 157.27 ± 170.82 pg/ml, respectively). PI3 presented similar levels between the two asthma groups (Figure 1F).

Furthermore, statistically significant differences were observed in the protein expression of SERPINB2, CHI3L1, IL-8, and POSTN when comparing severity subgroups (Figure 2). The protein expression of SERPINB2 among severe NA patients was higher than the protein expression of SERPINB2 in severe AA patients (0.13 ± 0.07 and 0.06 ± 0.02 RQ, respectively, *P* = 0.0367) (Figure 2B). As also observed in Figure 2B, the moderate-mild AA group showed significantly higher SERPINB2 expression than the severe AA group (0.11 ± 0.04 vs. 0.06 ± 0.02 RQ, respectively, *P* = 0.0367). For CHI3L1 there were significant differences between the severe NA group and the severe AA group (*P* = 0.0021) (Figure 2C), with lower CHI3L1 levels in NA patients. Significant differences were found when comparing the levels of IL-8 in moderate-mild NA patients to moderate-mild AA patients (*P* = 0.0433) (Figure 2D), with lower levels found in AA patients. In Figure 2G, the protein levels of POSTN in serum are shown according to the severity of asthma. The levels of POSTN were higher in the severe NA patients when compared to the severe AA patients (*P* = 0.0468).

Though PHLDA1, IL-10, and PI3 did not present significant differences in protein levels between the severity subgroups (Figure 2), IL-10 showed higher levels in severe asthma patients (severe NA group: 200.60 ± 219.85 pg/ml and moderate-mild NA group: 105.27 ± 79.62 pg/ml; severe AA group: 211.49 ± 193.94 pg/ml and moderate-mild AA group 168.11 ± 136.13 pg/ml) (Figure 2E). PHLDA1 had the highest levels in the two NA severity subgroups, especially when patients had a moderate-mild diagnosis [severe NA (*n* = 3): 0.05 ± 0.07 RQ, moderate-mild NA (*n* = 2): 0.08 ± 0.10 RQ, severe AA (*n* = 3): 0.03 ± 0.04 RQ, moderate-mild AA (*n* = 3): 0.02 ± 0.01 RQ] (Figure 2A), although this data needs to be confirmed in a bigger population. PI3 had similar protein levels in all severity subgroups (severe NA: 6225.03 ± 1999.11 pg/ml, moderate-mild NA: 5824.14 ± 2624.29 pg/ml, severe AA: 6390.10 ± 2226.27 pg/ml, moderate-mild AA: 6784.24 ± 2311.30 pg/ml) (Figure 2F).

### ROC Curve Analysis of Protein Expression

When comparing AUC values between the NA group and the AA group (Table 3), interesting biomarkers were seen in patients with severe asthma. The protein expression of CHI3L1 and SERPINB2 were good candidates for discriminating severe NA from severe AA subjects (AUC value: 0.82 and 0.78, respectively). SERPINB2 (AUC value: 0.77) was a good candidate for distinguishing disease severity in the AA group. IL-8 was also classified as good for differentiating severe AA patients from moderate-mild AA patients (AUC value: 0.82) and, as described by Baos et al. (20), also when discriminating severe NA from moderate-mild NA (AUC value: 0.76). For all other comparisons, the AUC values of the individual proteins studied were insufficient for a good classification (Table 3). Consequently, the ROC curve analysis was determined by combining the protein expression of two (Table 3) and three (Table 4) biomarkers. There was an improvement in sensitivity and specificity when several biomarkers were combined in all of the comparisons. It is worth highlighting the presence of POSTN in many of the combinations. The combination of two or three biomarkers gave combinations with AUC values over 0.75, meaning that good, very good, or excellent test were found when discriminating the two asthma groups and their severity.

**Table 3:** Receiver operating characteristic (ROC) curve analyses of the protein expression combining two biomarkers.

	**CHI3L1**	**IL-10**	**IL-8**	**PI3**	**POSTN**	**PHLDA1**	**SERPINB2**
**(A) TOTAL NA GROUP COMPARED TO TOTAL AA GROUP**							
CHI3L1	0.74	0.73	**0.82**	0.75	**0.76**	0.43	**0.83**
IL-10	–	0.45	0.71	0.46	**0.87**	ND	ND
IL-8	–	–	0.68	0.67	**0.76**	ND	**0.82**
PI3	–	–	–	0.57	0.67	0.6	0.69
POSTN	–	–	–	–	0.62	0.7	0.75
PHLDA1	–	ND	ND	–	–	0.4	**0.80**
SERPINB2	–	ND	–	–	–	–	0.64
**(B) SEVERE NA GROUP COMPARED TO SEVERE AA GROUP**							
CHI3L1	**0.82**	0.69	**0.86**	**0.83**	**0.86**	ND	ND
IL-10	–	0.49	ND	0.68	**0.94**	ND	ND
IL-8	–	ND	0.63	0.69	**0.86**	ND	ND
PI3	–	–	–	0.48	0.69	ND	**0.92**
POSTN	–	–	–	–	0.69	ND	ND
PHLDA1	ND	ND	ND		ND	ND	ND
SERPINB2	ND	ND	ND	–	ND	ND	**0.78**
**(C) MODERATE-MILD NA GROUP COMPARED TO MODERATE-MILD AA GROUP**							
CHI3L1	0.66	0.60	**0.80**	0.63	0.72	ND	0.72
IL-10	–	0.60	ND	0.68	**0.82**	ND	ND
IL-8	–	ND	0.73	**0.82**	**0.82**	ND	ND
PI3	–	–	–	0.62	0.67	ND	0.68
POSTN	–	–	–	–	0.53	ND	0.40
PHLDA1	ND	ND	ND	ND	ND	ND	ND
SERPINB2	–	ND	ND	–	–	ND	0.60
**(D) SEVERE AA GROUP COMPARED TO MODERATE-MILD AA GROUP**							
CHI3L1	0.50	0.47	**0.82**	0.58	0.50	ND	**0.90**
IL-10	–	0.46	ND	0.65	0.62	ND	ND
IL-8	–	ND	**0.82**	**0.78**	**0.84**	ND	ND
PI3	–	–	–	0.56	0.54	ND	**0.87**
POSTN	–	–	–	–	0.52	ND	**0.93**
PHLDA1	ND	ND	ND	ND	ND	ND	ND
SERPINB2	–	ND	ND	–	–	ND	**0.77**

*Area under the curve values appearing in bold are >0.75 (good, very good, or excellent results). ND: not determined due to a lack of data. Cells with a dash refer to a comparison already shown in another cell of this table. Entries in gray indicate the individual ROC curve analysis of the protein expression of the seven biomarkers. NA, nonallergic asthma; AA, allergic asthma*.

**Table 4:** Receiver operating characteristic (ROC) curve analysis of the protein expression combining three biomarkers.

**Combination of biomarkers**	**NA vs. AA AUC value**	**MM NA vs. MM AA AUC value**	**S NA vs. S AA AUC value**
**(A) NA GROUP COMPARED TO AA GROUP**			
CHI3L1 + IL-10 + IL-8	**0.76**	ND	ND
CHI3L1 + IL-10 + PI3	0.71	0.68	0.72
CHI3L1 + IL-10 + POSTN	**0.92**	**0.90**	ND
CHI3L1 + IL-8 + PI3	**0.82**	0.71	**0.88**
CHI3L1 + IL-8 + POSTN	**0.88**	ND	**0.96**
CHI3L1 + PI3 + POSTN	**0.80**	**0.80**	**0.86**
CHI3L1 + PI3 + PHLDA1	0.63	ND	ND
CHI3L1 + PI3 + SERPINB2	**0.84**	0.72	ND
CHI3L1 + POSTN + PHLDA1	**0.77**	ND	ND
CHI3L1 + POSTN + SERPINB2	**0.85**	0.68	ND
CHI3L1 + PHLDA1 + SERPINB2	**0.84**	ND	ND
IL-10 + IL-8+PI3	**0.82**	ND	ND
IL-10 + IL-8+POSTN	**0.87**	ND	ND
IL-10 + PI3 + POSTN	**0.87**	**0.82**	**0.94**
IL-8 + PI3 + POSTN	**0.76**	**0.84**	**0.86**
IL-8 + PI3 + SERPINB2	**0.80**	ND	ND
IL-8 + POSTN+SERPINB2	**0.82**	ND	ND
PI3 + POSTN + PHLDA1	0.67	ND	ND
PI3 + POSTN + SERPINB2	**0.77**	0.64	ND
PI3 + PHLDA1+ SERPINB2	**0.84**	ND	ND
**Combination of biomarkers**		**AUC value**
**(B) SEVERE AA GROUP COMPARED TO MODERATE-MILD AA GROUP**			
CHI3L1 + IL-10 + PI3		0.65
CHI3L1 + IL-10 + POSTN		0.63
CHI3L1 + IL-8 + PI3		**0.82**
CHI3L1 + IL-8 + POSTN		**0.84**
CHI3L1 + PI3 + POSTN		0.56
CHI3L1 + PI3 + SERPINB2		**0.90**
CHI3L1 + POSTN+ SERPINB2		**0.93**
IL-10 + PI3 + POSTN		0.74
IL-8 + PI3 + POSTN		**0.82**
PI3 + POSTN + SERPINB2		**0.93**

*Numbers in bold denote area under the curve (AUC) values >0.75, which are considered good (AUC: 0.76–0.90), very good (AUC: 0.91–0.97), or excellent (AUC: 0.98–1) results. NA, nonallergic asthma; AA, allergic asthma; MM, group of subjects with moderate-mild asthma; S, group of subjects with severe asthma*.

To summarize, we ranked the best biomarkers or combination of biomarkers by their ability to discriminate each condition analyzed; all had a predictive accuracy that was at least good (AUC > 0.75) and a 95% CI of between 0.70 and 1. These rankings are shown in Table 5. Briefly, the combinations of IL-10 and POSTN, CHI3L1 with IL-10 and POSTN, or CHI3L1 with IL-8 and POSTN are proposed as good or very good sets of biomarkers to differentiate between the two types of asthma. For severity discriminations, IL-10 + POSTN, PI3 + SERPINB2, CHI3L1 + POSTN, and CHI3L1 + IL-8 + POSTN may be very good candidates for differentiating severe asthma diagnosis within the NA and AA groups; and CHI3L1 with IL-10 and POSTN may be useful for distinguishing between moderate-mild asthma patients (NA vs. AA). Furthermore, the combination of the protein expressions of POSTN and SERPINB2 was observed as a very good discriminator for the severity of AA patients (severe AA vs. moderate-mild AA). Added to this, and as found previously (20), POSTN, and SERPINB2, PI3 combined with POSTN and SERPINB2, or CHI3L1 with IL-8 and POSTN were found to be good, very good, and excellent biomarkers, respectively, as concerns their capacity to distinguish patients with severe NA from moderate-mild NA.

**Table 5:** Ranking of the best individual and combined proteic biomarkers for each discrimination.

	**AUC value (95% CI)**	**Threshold**
**(A) BIOMARKERS ABLE TO DISCRIMINATE NA PATIENTS FROM AA PATIENTS**		
CHI3L1	0.74 (0.61–0.88)	20,202
IL-10 + POSTN	**0.87 (0.74–1.00)**	**457.4, 22,785**
CHI3L1 + SERPINB2	0.83 (0.64**–**1.00)	20,202, 0.116
CHI3L1 + IL-8	0.82 (0.64**–**0.99)	20,202, 221
IL-8 + SERPINB2	0.82 (0.58**–**1.00)	221, 0.116
PHLDA1 + SERPINB2	0.80 (0.50**–**1.00)	0.012, 0.116
IL-8 + POSTN	0.76 (0.57**–**0.94)	221, 22,785
CHI3L1 + IL-10 + POSTN	**0.92 (0.81–1.00)**	**20,202, 457.4, 22,785**
CHI3L1 + IL-8 + POSTN	**0.88 (0.76–1.00)**	**20,202, 221, 22,785**
PI3 + PHLDA1 + SERPINB2	0.84 (0.57**–**1.00)	5,126, 0.012, 0.166
CHI3L1 + PI3 + POSTN	0.80 (0.68**–**0.91)	20,202, 5,126, 22,785
**(B) BIOMARKERS ABLE TO DISCRIMINATE SEVERE NA PATIENTS FROM SEVERE AA PATIENTS**		
CHI3L1	0.82 (0.67**–**0.98)	20,202
SERPINB2	0.78 (0.47**–**1.00)	0.11
IL-10 + POSTN	**0.94 (0.83–1.00)**	**184.6, 22,785**
PI3 + SERPINB2	**0.92 (0.76–1.00)**	**5,758, 0.11**
CHI3L1 + POSTN	**0.86 (0.72–1.00)**	**20,202, 22,785**
IL-8 + POSTN	0.86 (0.65**–**1.00)	122, 22,785
CHI3L1 + IL-8	0.86 (0.64**–**1.00)	20,202, 122
CHI3L1 + IL-8 + POSTN	**0.96 (0.86–1.00)**	**20,202, 122, 22,785**
**(C) BIOMARKERS ABLE TO DISCRIMINATE MODERATE-MILD NA PATIENTS FROM MODERATE-MILD AA PATIENTS**		
IL-10 + POSTN	0.82 (0.59**–**1.00)	167.9, 24,658
IL-8 + POSTN	0.82 (0.58**–**1.00)	221, 24,658
IL-8 + PI3	0.82 (0.53**–**1.00)	221, 5,028
CHI3L1 + IL-8	0.80 (0.51**–**1.00)	20,227, 221
CHI3L1 + IL-10 + POSTN	**0.90 (0.72–1.00)**	**20,227, 167.9, 24,658**
CHI3L1 + PI3 + POSTN	0.80 (0.64**–**0.97)	20,227, 5,028, 24,658
**(D) BIOMARKERS ABLE TO DISCRIMINATE SEVERE AA PATIENTS FROM MODERATE-MILD AA PATIENTS**		
IL-8	0.82 (0.58**–**1.00)	17
SERPINB2	0.77 (0.44–1.00)	0.095
POSTN + SERPINB2	**0.93 (0.78–1.00)**	**19,119, 0.095**
CHI3L1 + SERPINB2	0.90 (0.69**–**1.00)	24,144, 0.095
PI3 + SERPINB2	0.87 (0.59**–**1.00)	5,524, 0.095
IL-8 + POSTN	0.84 (0.62**–**1.00)	17, 19,119

*Summary of the best options for discriminating each condition obtained from the ROC curve analysis of protein expression from each of the seven biomarkers, either alone or in combination. AUC value: area under the curve. 95% CI, 95% confidence interval. Threshold means the protein levels of each biomarker able to discriminate each condition, with the AUC indicated. Options with the best statistical power (95% CI between 0.70 and 1) appear in bold. NA, nonallergic asthma; AA, allergic asthma*.

## Discussion

While the currently used therapeutic approach for asthma is effective in some patients, there remains an unmet need for a wider ray of treatment options, especially in uncontrolled severe asthma. Some studies propose specific approaches for the management of severe, difficult-to-treat asthma based on specific phenotype characteristics and biomarkers (28). The allergic asthma mediated by type T2 inflammation (T2 endotype) has been the center of attention in the search for biomarkers and for the development of therapeutic drugs used for managing eosinophilic inflammation. Comparatively, however, the asthma with non-T2 mediated immune response (non-T2 endotype) has received scant attention (29). Therefore, insufficient efforts have been devoted to this type of asthma, and biologically targeted therapies are an underdeveloped field.

The long-term aim is to establish an approach that modifies the disease, and this could be achieved through the development of drugs that target specific inflammatory pathways within asthma pathogenesis (30). Nevertheless, it is important to identify patients with a higher likelihood of responding to this kind of therapies. If proven effective, predictive biomarkers would mark a change in the classification and conventional treatment of asthma, putting an end to the “one size fits all” approach (31). Technologies of massive analysis, or *omics*, such as transcriptomics, proteomics, lipidomics, and metabolomics, are becoming very useful in the discovery of new biomarkers and in discriminating phenotypes in blood, sputum, bronchoalveolar lavage, or tissue samples derived from affected organs. Omics been proven to be effective in improving the classification of asthma and respiratory allergy, and therefore, in guiding targeted therapies. Peters et al. described a gene expression network analysis in sputum cells that can reveal airway immune-cell dysfunction in asthma associated with three endotypes: T2-low, T2-high, and T2-ultra high (32). Even so, the wealth of information provided on asthma and respiratory allergy by this type of complex studies is still underexploited (33, 34).

Further, this information must be contrasted and validated in samples that are easy to process. Following this idea, this work seeks to validate some of these results in PBMCs, specifically of 94 genes described previously as possible biomarkers for discrimination of asthma and respiratory allergy phenotypes (21). Given the results of this genetic study, the most relevant candidates were chosen to be assessed at the protein level. The biomarkers found to be important at the genetic level for distinguishing asthma and its severity were studied, and as the goal is to find easy-to-process biomarkers, simple techniques such as ELISA or Western blot were used. Recently, we reported on a group of genes and proteins that are differently expressed in peripheral blood samples from patients with nonallergic asthma (T2-low) compared to healthy control subjects, linking some of these genes to disease severity (20). In this project, the relevance of the gene and protein expression of potential markers has been explored in depth by studying differences in protein levels and through the analysis by ROC curves using their individual and combined expression, with the goal of demonstrating their capacity to discriminate between two asthma phenotypes (nonallergic and allergic) and disease severity using peripheral samples. These biomarkers were as follows: CHI3L1, IL-8, IL-10, SERPINB2, PHLDA1, PI3, and POSTN. The functional and pathological implications of these biomarkers in asthma have been discussed previously (20, 21). Briefly, *CHI3L1* (chitinase 3-like 1) encodes a glycoprotein member of the glycosyl hydrolase 18 family or YKL-40. This protein has been extensively studied in relation with asthma, and recently proposed not only as a potentially useful biomarker for identification of severity, but also a potential therapeutic target (35). *IL-8* is a member of the CXC chemokine family and one of the major mediators of the inflammatory response. It is a chemo-attractant for neutrophils and very important for many neutrophil functions. This is one of the few biomarkers proposed for asthma mediated by non type-2 inflammation. In fact, the use of CXCR2 (the high-affinity receptor of IL8) antagonist is one of the few targeted therapies proposed for non type-2 inflammation, although until now no study could demonstrate clinical effectiveness (36, 37). IL-10 is a cytokine with pleiotropic effects, considered as one of the main regulatory cytokines (38) and extensively associated with asthma and allergy diseases (39). *PHLDA1 (pleckstrin homology-like domain, family A, member 1)* has only been associated with asthma diseases by our group (20, 21, 24). *PI3* (peptidase inhibitor 3 skin-derived) encodes an elastase-specific inhibitor (elafin) that inhibits serine proteases, such as human neutrophil elastase and proteinase 3, to prevent excessive damage during inflammation. It has been postulated as protective for asthma (40). Finally, *SERPINB2*, a member of the serine protease inhibitor family, was mainly associated with type 2 inflammation together with *POSTN* (periostin) and *CLCA1* in airway epithelial cells upon IL13 stimulation (41).

Here, we propose the use of individual biomarkers and where discrimination is not optimal, we argue in favor of using sets of biomarkers for better patient characterization. Therefore, the gene expression of *CPA3* and *IL-8* were found to be important when differentiating between the two asthma phenotypes as well as the severity of moderate-mild asthma (Table 2). To discriminate patients with severe disease in the two asthma groups, *PI3* (AUC value: 0.78) was seen to be a good biomarker (Table 2). Approaches using single parameters are still important in the process of discovering biomarkers. Thus, existing and newly identified biomarkers should be integrated to reinforce the clinical value of stratifying asthmatic phenotypes and endotypes (6).

At the protein level, both individually and in combination, we have detected biomarkers with high potential (Tables 3, 4). With strict criteria (best AUC value and 95% CI), the panels of biomarkers that best differentiated our two groups and their severity were as follows (Table 5): the combination of CHI3L1 +IL-10 +POSTN was very good for differentiating the NA from the AA group, but also good for comparing moderate-mild NA patients from moderate-mild AA subjects; CHI3L1 +IL-8 + POSTN was very effective in discriminating severe asthma within the NA group from the AA group [AUC value of 0.96 (0.86–1.00), Threshold: 20,202, 122, 22,785, Table 5]; and lastly, the combination of POSTN and SERPINB2 is proposed as a very useful test for comparing severe vs. moderate-mild AA subjects. For patients with NA and different severities (severe vs. moderate-mild), the combination of CHI3L1 + IL-8 + POSTN has been previously proposed (20) as an excellent test [AUC value of 0.98 (0.92–1.00), Threshold: 18,500, 841, 17,419]. The same combination of biomarkers could be useful for discriminating distinct groups of patients, given that threshold values and the AUC are different in each case.

In summary, these results mark a starting point for the improvement of asthma diagnosis and treatment. Though encouraging, our results have several limitations that should be addressed. Interesting would be to analyze the correlation of expression between the peripheral sample levels and the target-tissue of the disease. Indeed, the stability of these biomarkers in time and their possible modification with medication should be analyzed in longitudinal studies, in bigger populations, and by different research groups to corroborate their validity and reproducibility. Nowadays, overlap between different groups of endotypes and the variety in the effects of therapy remains substantial, and by further defining the pathogenesis of asthma we will be able to clarify these confusing differences. Our results may help in this effort, given that we contribute new biomarkers to the few that currently exist associated to the nonallergic asthma and its severity. Moreover, we propose alternatives to the biomarkers already used in clinical practice.

## Ethics Statement

Written informed consent in accordance with the Declaration of Helsinki was obtained from each subject. Ethical approval for the study was obtained from the ethical and research committees of the participating hospitals.

## Author Contributions

SB and BC have worked on all steps of the project, i.e., study design, experimental work, results discussion, and manuscript drafting. DC, LC-J, and MdP collaborated in drafting the manuscript. JS, CP, JQ, and FF performed patient selection and collaborated in the design of the study. CL collaborated in the study design, results discussion, and in drafting the manuscript.

### Conflict of Interest Statement

The data published in this report are protected (Patent Number: PCT/ES2018/070515). The authors declare that the research was conducted in the absence of any commercial or financial relationships that could be construed as a potential conflict of interest.
